# Relationship Between Muscle Tone and Elasticity: Simultaneous Quantitative Assessment Using Train-of-Four Monitoring and Continuous Shear Wave Elastography During Anesthesia Induction—A Prospective Observational Study

**DOI:** 10.3390/diagnostics15030293

**Published:** 2025-01-26

**Authors:** Hitoshi Shitara, Tadanao Hiroki, Ren Koda, Tsuyoshi Sasaki, Masataka Kamiyama, Ryosuke Miyamoto, Kurumi Nakase, Fukuhisa Ino, Takuma Kachi, Rie Mieda, Chizu Aso, Yoshiki Yamakoshi, Shigeru Saito, Hirotaka Chikuda

**Affiliations:** 1 Department of Orthopaedic Surgery, Graduate School of Medicine, Gunma University, 3-39-15 Showa-machi, Maebashi-shi 371-8511, Japan; tsasaki@gunma-u.ac.jp (T.S.); m-kamiyama@gunma-u.ac.jp (M.K.); r-miyamoto@gunma-u.ac.jp (R.M.); walnut0523@gmail.com (K.N.); inofuku423@gmail.com (F.I.); takuman21@gmail.com (T.K.); chikuda-tky@umin.ac.jp (H.C.); 2 Department of Anesthesiology, Graduate School of Medicine, Gunma University, 3-39-22 Showa-machi, Maebashi-shi 371-8511, Japan; t-hiroki@gunma-u.ac.jp (T.H.); rmieda@gunma-u.ac.jp (R.M.); chizua@gunma-u.ac.jp (C.A.); shigerus@gunma-u.ac.jp (S.S.); 3 Graduate School of Science and Technology, Gunma University, 1-5-1 Tenjin-cho, Kiryu-shi 376-8515, Japan; koda@gunma-u.ac.jp (R.K.); yamakoshi@gunma-u.ac.jp (Y.Y.); 4 Institute of Innovative Research, Institute of Science Tokyo, 4259 R2-25, Nagatsuta-cho, Midori-ku, Yokohama-shi 226-8503, Japan

**Keywords:** shear wave elastography (SWE), train-of-four (TOF) monitoring, anesthesia induction, vastus lateralis

## Abstract

**Background/Objectives**: Non-invasive evaluations of muscle elasticity and tone are crucial in musculoskeletal medicine. Shear wave elastography (SWE) provides quantitative assessments of muscle elasticity, whereas train-of-four (TOF) monitoring measures muscle tone during neuromuscular blockades. This study investigated the relationship between muscle elasticity and tone during anesthesia induction using continuous SWE (C-SWE) and TOF monitoring. **Methods**: Fifteen patients who underwent general anesthesia with rocuronium were recruited. The muscle elasticity of the vastus lateralis was assessed using C-SWE, and muscle tone was evaluated using TOF monitoring. Measurements were taken at two time points: before rocuronium administration and during complete muscle relaxation, confirmed by the TOF ratio (TOF = 0). Statistical analyses were performed using paired *t*-tests and correlation analyses. **Results**: Data from 11 patients were analyzed. The mean shear wave velocity (SWV) decreased significantly from 5.0 ± 0.4 m/s before rocuronium administration to 3.1 ± 0.3 m/s during complete relaxation (*p* < 0.0001). A significant negative correlation was observed between the baseline SWV and the degree of reduction in the SWV (r = −0.728, *p* = 0.011). No significant correlations were found between SWV and demographic factors such as age, sex, height, or body mass index. **Conclusions**: C-SWE and TOF monitoring are reliable and sensitive methods for evaluating muscle elasticity and tone during general anesthesia. This study highlights the interplay between muscle tone and elasticity, suggesting that muscles with a higher baseline tone exhibit a greater reduction in elasticity after relaxation. These findings have implications for improving intraoperative muscle function assessments and advancing the application of C-SWE in clinical practice.

## 1. Introduction

In the field of musculoskeletal medicine, non-invasive measurement of the elasticity of biological tissues, such as skeletal muscles and tendons, is gaining attention as a valuable quantitative evaluation method. Recent advancements in ultrasound elastography have introduced innovative techniques for assessing tissue elasticity, and shear wave elastography (SWE) has emerged as a promising tool for this purpose. SWE enables quantitative and reproducible measurements of the mechanical properties of muscles and tendons, showing potential for applications in the diagnosis, staging, and monitoring of various musculoskeletal conditions [1,2,3,4].

Among the various approaches for assessing tissue elasticity, one notable method is acoustic radiation force impulse (ARFI) imaging, which utilizes ultrasonic waves to generate shear waves within the tissue [5,6,7]. This technique employs high-intensity ultrasonic pulses to generate acoustic radiation force impulses. However, this method has certain limitations. For instance, the use of intense ultrasonic waves may cause undesirable increases in temperature, particularly when the bones or similar structures are near the focal point [8,9,10]. In addition, the detection of shear waves typically requires specialized high-frame-rate ultrasound imaging devices, resulting in increased system complexity and costs.

**Figure 1:**
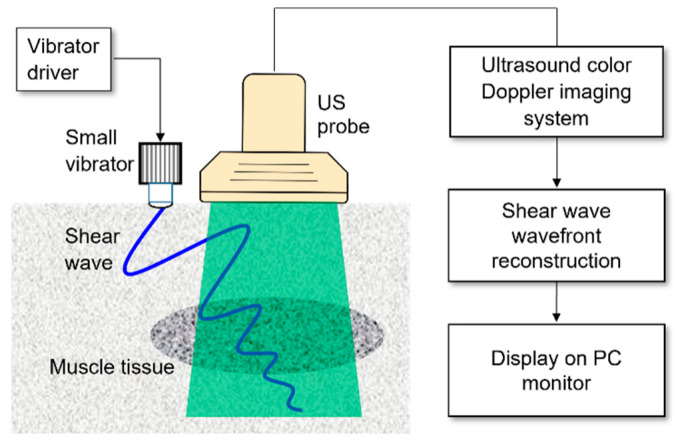
Schematic diagram of C-SWE. Shear waves are generated by a small vibrator placed on the surface of the body. Ultrasound signals are received by an ultrasound probe to detect the propagation of shear waves. In C-SWE, a color Doppler signal processing unit is used to reconstruct the shear wavefront. The color Doppler images are imported into a PC via a video capture device, and the shear wavefront is reconstructed on the PC. The reconstructed wavefront is displayed in real time on the PC monitor.

To address these issues, a novel approach for estimating the shear wave velocity (SWV) using mechanical excitation has been extensively studied [11,12,13]. Continuous shear wave elastography (C-SWE), developed by Yamakoshi et al. [14,15], represents a significant advancement. The C-SWE system visualizes the wavefront and velocity of the shear waves generated by a compact vibrator measuring only a few centimeters in height and width (Figure 1). One of the key advantages of C-SWE is its ability to leverage color Doppler images for processing without requiring modifications to conventional ultrasound equipment equipped with color Doppler functionalities. By simply importing these color Doppler images into a computer for processing, the system enables quantitative imaging of tissue stiffness. This makes the technology both cost-effective and versatile because it can be implemented in various ultrasound devices, including portable tablet-based systems. Consequently, C-SWE is suitable for a wide range of environments, including medical examination rooms, bedside settings, physical therapy settings, and sporting events. Additionally, the system allows for real-time observation of shear wave propagation. Unlike techniques that rely on acoustic radiation to generate shear waves, C-SWE employs mechanical excitation to ensure a high level of safety in living tissues. This method allows for the quantitative visualization of shear waves with minimal additional equipment, offering a simple yet effective solution for assessing tissue elasticity.

The relationship between muscle tone and elasticity is complex, as both properties contribute to muscle function but represent distinct physiological characteristics. Muscle tone refers to the continuous and passive partial contraction of muscles or the muscle’s resistance to passive stretching in a resting state [16]. Neural factors, including reflex excitability and the intrinsic properties of contractile elements, primarily influence it [17]. Muscle elasticity refers to the ability of a muscle to return to its original length after stretching, reflecting the viscoelastic properties of the musculotendinous unit [17].

When evaluating living organisms in real time, muscle tone is influenced by various factors, including control by the higher central nervous system, spinal cord, and peripheral nerves, as well as autonomic nervous system activity, psychological state, pain, and external stimuli, such as auditory, visual, and tactile inputs [18,19]. Consequently, muscle elasticity can change dynamically over time. This variability presents a significant challenge in regard to skeletal muscle assessment, as it becomes difficult to discern whether the observed changes in muscle elasticity are due to actual physiological variations or the influence of data variability related to reproducibility or validity, particularly in the absence of standardized reference data due to individual differences. In addition, the relationship between muscle tone and elasticity remains unclear.

To address this issue, it is necessary to evaluate skeletal muscles under conditions in which external stimuli are minimized, which induces uniform changes in muscle elasticity, and muscle tone can be objectively measured using other methods. In this study, we considered that this issue could be resolved by objectively evaluating muscle tone through muscle relaxation monitoring using an electromyography (EMG)-based neuromuscular monitor and simultaneously assessing muscle elasticity using C-SWE under general anesthesia with the administration of muscle relaxants. This study aimed to determine the relationship between the degree of muscle relaxation, as assessed by monitoring muscle relaxation, and muscle elasticity, as assessed by C-SWE, before and after the use of muscle relaxants during general anesthesia. We hypothesized that muscle relaxant administration would be accompanied by a decrease in muscle tone, which was confirmed by neuromuscular monitoring, and a corresponding decrease in muscle elasticity, which could be detected using C-SWE. By elucidating the relationship between muscle tone and elasticity, it is possible to discuss the reproducibility and validity of C-SWE as a reliable method for assessing skeletal muscle characteristics in various clinical settings and potentially improving the management of critical illnesses and rehabilitation processes. The findings from this study hold considerable potential for advancing clinical practice. First, the simultaneous assessment of muscle tone and elasticity during general anesthesia provides a novel tool for optimizing perioperative muscle management which could improve intubation conditions, enhance patient safety, and minimize post-surgical complications. Second, elucidating the relationship between muscle tone and elasticity offers valuable insights into managing neuromuscular disorders such as spasticity and muscular dystrophy, facilitating tailored rehabilitation and therapeutic strategies. Third, this study contributes to the validation and standardization of continuous shear wave elastography (C-SWE) as a reliable, non-invasive method for assessing muscle characteristics, supporting its integration into diverse clinical environments. Lastly, the ability to quantify muscle elasticity in real time enhances the precision of neuromuscular management, particularly during anesthesia and in conditions involving altered muscle tone. By bridging the gap between muscle tone and elasticity assessment, this research provides a foundation for improving patient outcomes across perioperative care, rehabilitation, and broader forms of musculoskeletal medicine.

## 2. Materials and Methods

### 2.1. Participants

We recruited patients who visited the orthopedic clinic at Gunma University Hospital between March and May 2023. The inclusion criteria were as follows: age ≥ 18 years; scheduled to undergo surgery under general anesthesia in the orthopedic surgery department; good general health with a Performance Status (PS) score ≤ 2; planning to receive muscle relaxation monitoring and administration of rocuronium bromide as a muscle relaxant; no diseases causing muscle atrophy or degeneration at the measurement site; and no history or current diagnosis of psychiatric or neurological disorders.

Patients were excluded if they were pregnant, had a history of hypersensitivity to rocuronium bromide or other bromides, or had been diagnosed with myasthenia gravis or myasthenic syndrome accompanied by hypersensitivity to sugammadex sodium. Patients who used pacemakers were excluded from the study.

All participants provided written informed consent before their inclusion in the study.

### 2.2. Clinical Trial Registration

This single-center clinical trial was approved by the Institutional Review Board of Gunma University Hospital (approval no. IRB2022-033, 6 October 2022) and registered prior to patient enrolment in the Japan Registry of Clinical Trials (jRCT; identifier: jRCTs032220472, 28 November 2022).

### 2.3. Experimental Protocol in Induction of General Anesthesia

The patients were positioned supine on the operating table. Anesthesia induction was performed as follows: After 2 min of oxygenation, induction was initiated with the administration of propofol at a dose of 0.5–1.5 mg/kg and remifentanil at 0.2–0.3 µg/kg/min. Once the patient fell asleep, 5% sevoflurane or desflurane was administered, and the muscle relaxation monitor was calibrated. After confirming that the train-of-four (TOF) response could be observed, rocuronium was administered at a dose of 0.4 mg/kg, which is a relatively low dose because we wanted to detect slow changes in muscle relaxation. After verifying that the TOF ratio had reached and stabilized at 0, an additional 0.4 mg/kg of rocuronium was administered to achieve more profound muscle relaxation. Tracheal intubation was performed 1 min later, and positive mechanical ventilation was initiated. Muscle elasticity was continuously evaluated using C-SWE from the time the patient fell asleep, and the TOF ratio was stabilized at 0. For analysis, data were extracted at two time points: (1) before rocuronium administration and (2) when complete muscle relaxation confirmed that the TOF ratio stabilized at zero. The primary endpoint was the difference in muscle elasticity before rocuronium administration and the state of complete muscle relaxation, while the secondary endpoint was the correlation between muscle elasticity before rocuronium administration and the state of complete muscle relaxation.

### 2.4. Quantitative Neuromuscular Monitoring with the Train-of-Four Ratio (TOF)

Before the induction of general anesthesia, EMG AF-201P (Nihon-Kohden, Tokyo, Japan) was applied to the abductor digiti minimi (ADM) muscle of the arm, which was not on the surgical side. Following appropriate skin preparation, disposable surface electrodes (NM-345Y; Nihon-Kohden, Tokyo, Japan) were positioned over the ADM muscle [20]. After anesthesia induction, TOF stimulation was applied at a frequency of 2 Hz for 2 s, then repeated every 15 s [21], following automated calibration of the supramaximal current and responses.

### 2.5. Measurement of the Shear Wave Velocity (SWV) of the Vastus Lateralis

The intrarater validity and reliability of this method have been previously established [22]. The SWV was measured in the right vastus lateralis muscle using a multifrequency 11 MHz linear-array transducer (LOGIQ S8; GE Healthcare, Chicago, IL, USA) and a custom-made vibrator (specifications: frequency 60–150 Hz, vibration amplitude, 1 mm or less) to continuously generate shear waves. An elastic attachment fabricated using a 3D printer (Anycubic Kobra 2 Neo, Shenzhen Anycubic Technology Co., Ltd., Shenzhen, China) was used to maintain the ultrasonic array transducer at a constant distance from the vibrator. All measurements were performed by a single orthopedic surgeon (H.S.) with 15 years of experience in the field of musculoskeletal ultrasound.

The vastus lateralis muscle was selected because of its anatomical accessibility, large size, and clinical significance. Its superficial location and well-defined landmarks facilitated reliable shear wave elastography measurements. Moreover, as it is a critical muscle for locomotion and postural maintenance, understanding its tone and elasticity under neuromuscular blockade provides clinically meaningful insights.

**Figure 2:**
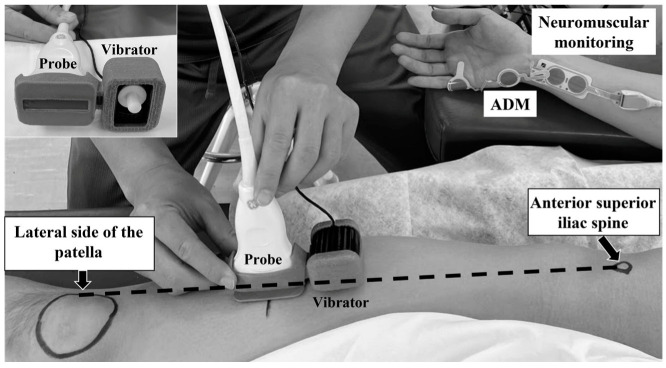
Neuromuscular monitoring and positioning of the US probe and vibrator during measurement of the SWV of the right vastus lateralis muscle. ADM: abductor digiti minimi muscle.

The subjects lay supine on a bed. According to the SENIAM (Surface Electromyography for the Non-Invasive Assessment of Muscles) guidelines (http://www.seniam.org) [23], the measurement point was determined by marking two-thirds of the distance along the line from the anterior superior iliac spine to the lateral side of the patella. The probe of the ultrasound diagnostic device was positioned parallel to the long axis of the muscle fibers directly over this measurement point, with the excitation point of the vibrator being placed proximal to the probe (Figure 2).

**Figure 3:**
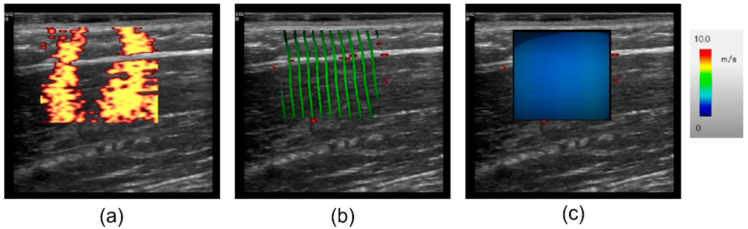
Example of a reconstructed shear wave map obtained by C-SWE. (**a**) Color Doppler image (power mode); (**b**) shear wave propagation map, (**c**) shear wave velocity map.

Before the measurement, the positions of the probe and excitation point were carefully adjusted to ensure that the shear waves propagated along the direction of the muscle fibers in the vastus lateralis, as observed in the shear wave wavefront images. Once the desired propagation pattern was achieved, the measurements were recorded continuously. The measurement with the most uniform shear wave wavefronts satisfying the previously described analysis conditions was automatically selected to determine the shear wave propagation speed (Figure 3). An SWV analysis was performed using a previously developed software that obtains the SWV by analyzing the binary pattern of shear wave propagation appearing on power Doppler images [24]. The frame rate and the ultrasound frequency of power in the Doppler mode were 29 fps and 4.2 MHz, respectively. The sizes of the region of interest (ROI) were 22.1 and 26.5 mm in the depth and horizontal directions, respectively. The system visualizes shear waves at 5.54 s/frame [24]. To improve the quantitative nature of the SWV measurements, high-quality images suitable for SWV measurements were extracted. We introduced the Q-factor, which is an index of the shear wave amplitude throughout the image, and the shear wave propagation direction index (SWDI), which is an index of the uniformity of the shear wave propagation direction [24]. In this analysis, the conditions adopted were Q-factor>0.5 and SWDI>80. In addition, extraction was performed based on the peak position of the shear wave complex amplitude. The SWV was measured until the train-of-four ratio (TOF) stabilized at zero.

### 2.6. Sample Size and Statistical Analysis

Sample size calculations were not performed because no study has measured changes in muscle elasticity under general anesthesia. The sample size was based on previous studies [14,25]; 11 participants were required and 15 were recruited in anticipation of 20% missing data.

All statistical analyses were performed using IBM SPSS Statistics software (version 26; IBM Japan, Tokyo, Japan), except for the post hoc power analysis.

All tests were two-sided, with a significance level of *p* < 0.05. We compared the difference in the SWV between the pre-rocuronium administration period and the time at a train-of-four (TOF) ratio of 0 using a paired *t*-test. A correlation analysis was performed between demographic data and the SWV.

Finally, the post hoc power analysis of correlation was performed using G*Power 3.1.9.7 to calculate the statistical power of this study.

## 3. Results

**Figure 4:**
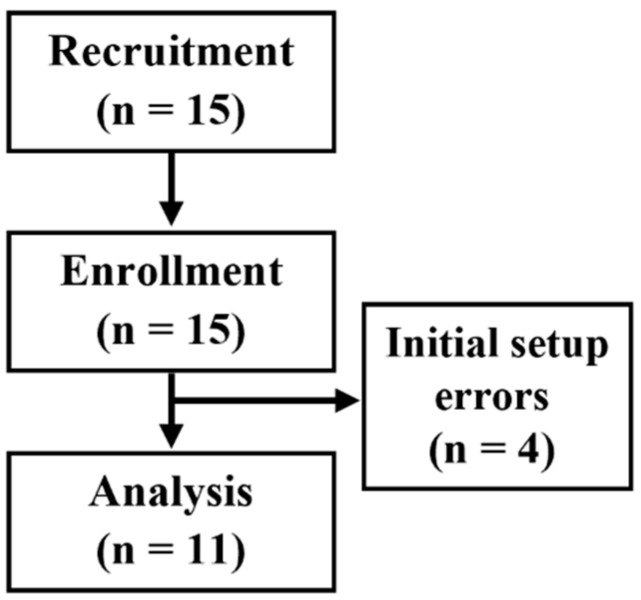
Patient flow diagram.

Fifteen patients who met the inclusion criteria were enrolled in this study. However, four patients were excluded from the analysis because of initial setup errors during the measurement. Initial setup errors refer to issues such as the predefined ROI for Doppler images not matching the depth of the target muscle. Although adjustments to the ROI were attempted during measurement, this deviation from the software’s constraints rendered the analysis unusable. Consequently, data from 11 patients were included in the final analysis (Figure 4).

The mean ± standard error of the mean (SEM) of the age, height, weight, and body mass index (BMI) of the cohort were 59.4 ± 4.6 years, 163.4 ± 2.0 cm, 70.1 ± 3.7 kg, and 26.3 ± 1.3 kg/m^2^, respectively. (Table 1)

**Figure 5:**
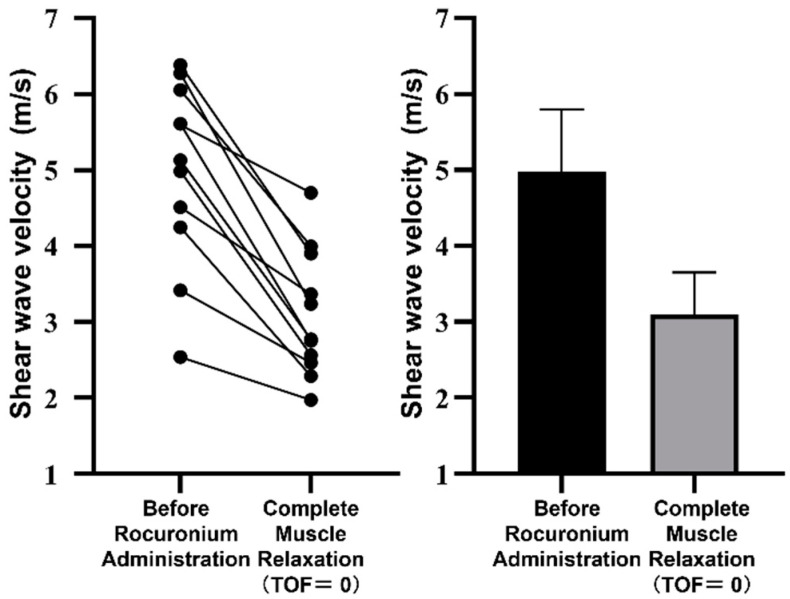
Changes in the shear wave velocity between the pre-rocuronium administration period and the time of complete muscle relaxation, that is, the train-of-four ratio (TOF) = 0. (**Left**) Individual changes in shear wave velocity. (**Right**) Mean values with error bars representing 95% confidence intervals.

The SWV at complete muscle relaxation was significantly decreased compared to the SWV before rocuronium administration (the mean ± SEM of SWV before rocuronium administration and complete muscle relaxation, that is, TOF = 0, were 5.0 ± 0.4 and 3.1 ± 0.3 m/s, respectively. *p* < 0.0001). The SWV in all participants decreased during complete muscle relaxation compared with before rocuronium administration (Figure 5).

**Figure 6:**
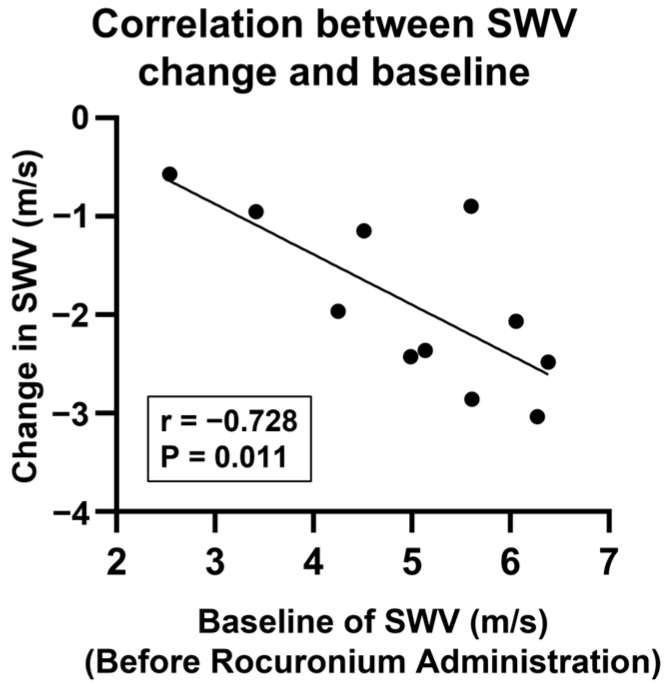
Correlation between the change in shear wave velocity and baseline. SWV: shear wave velocity. Change: (complete muscle relaxation − before rocuronium administration) in SWV.

There was no significant correlation between the demographic data and the SWV at any time point or change. (Table 2) The change in the SWV was significantly negatively correlated with the SWV before rocuronium administration, supporting the hypothesis that reduced muscle elasticity, as indicated by the SWV, corresponds to a decreased SWV (r = −0.728, *p* = 0.011) (Figure 6).

## 4. Discussion

This study demonstrated that the SWV of the right vastus lateralis muscle, measured using continuous shear wave elastography (C-SWE), was significantly decreased during complete muscle relaxation compared to that before rocuronium administration. Simultaneous TOF monitoring confirmed the depth of the neuromuscular blockade, enabling objective assessment of both muscle tone and elasticity. Additionally, higher muscle elasticity at baseline was significantly associated with a greater reduction in muscle elasticity after muscle relaxant administration. To the best of our knowledge, this is the first study to simultaneously evaluate electrophysiological changes and elasticity in muscles under general anesthesia, offering novel insights into the relationship between muscle tone and elasticity.

### 4.1. Relationship Between Muscle Tone and Elasticity

A previous study [17] assessed the viscoelastic properties of muscles using myotonometry and found that muscle stiffness, a component related to elasticity, was more responsive to interventions than muscle tone. Additionally, factors such as BMI correlate differently with muscle tone and elasticity. In a study of 172 healthy subjects (mean age, 26.00 ± 5.45 years), an increased BMI was associated with higher muscle tone and stiffness, particularly in the lower extremities, as well as reduced elasticity in the upper extremities [26]. In the present study, no significant association was observed between BMI and muscle elasticity. The small number of participants may have contributed to this discrepancy. These findings suggest that, although muscle tone and elasticity are interrelated, they are influenced by different factors and can respond differently to physiological changes and interventions. Understanding this relationship is crucial for the development of targeted therapeutic strategies for rehabilitation and clinical practice.

To the best of our knowledge, no study has simultaneously evaluated changes in muscle tone and elasticity. This study uniquely demonstrated a significant reduction in muscle elasticity during a complete neuromuscular blockade, underscoring the complex interactions between muscle tone and elasticity. To further understand the relationship between muscle tone and elasticity in more detail, future studies (regarding elements such as the temporal synchronization of SWV and TOF measurements) and a larger number of cases are needed.

### 4.2. Muscle Elasticity During an Anesthetic Induction

A previous study [27] examined the elasticity of the abdominal muscles using shear waves produced by acoustic pulses during anesthetic induction at three different time points: (1) while receiving only oxygen before remifentanil administration, (2) during remifentanil administration before anesthetic induction (opioid-induced rigidity), and (3) after anesthetic induction (complete muscle relaxation). At baseline, the SWV was 1.91 ± 0.51 m/s. During opioid-induced rigidity, the SWV was 2.24 ± 0.65 m/s, which was faster than the baseline value. At complete muscle relaxation without neuromuscular monitoring, the SWV was 1.61 ± 0.38 m/s, which was slower than the baseline. Similarly, our study found that the SWV of the vastus lateralis muscle was significantly reduced during complete muscle relaxation (3.1 ± 0.3 m/s), which was confirmed by TOF = 0 compared to before rocuronium administration (5.0 ± 0.4 m/s), supporting the hypothesis that reduced muscle elasticity corresponds to a decreased SWV. The fact that neuromuscular monitoring in this study confirmed complete muscle relaxation gives it an advantage over previous studies. This decrease in muscle elasticity during the neuromuscular blockade is consistent with the known physiological effects of non-depolarizing muscle relaxants.

In the present study, the period during remifentanil administration before anesthetic induction (opioid-induced rigidity) was not measured because we believe that the influence of remifentanil on muscle tone is minimal compared to the profound effect of rocuronium during complete muscle relaxation, as the dose of remifentanil we used (0.2–0.3 μg/kg/min) was substantially lower than the 0.5 mg/kg/min dose utilized in the previous study, at approximately 1/2000 of the previous amount [27]. Further research is required to fully understand the relationship between muscle tone and elasticity under various pharmacological conditions.

Interestingly, we observed a significant negative correlation between the baseline SWV and the degree of SWV reduction after relaxation. This suggests that muscles with a higher initial tone exhibit a greater reduction in elasticity following the administration of a muscle relaxant. Clinically, this finding is particularly relevant for tailoring muscle relaxant dosages in perioperative settings. Patients with a higher baseline muscle tone may require closer monitoring and individualized dosing to achieve adequate relaxation while minimizing the risk of under- or over-relaxation. Additionally, this observation supports the potential utility of C-SWE as a diagnostic tool for conditions characterized by altered muscle tone, such as spasticity or rigidity in neuromuscular disorders. By detecting subtle changes in muscle elasticity, C-SWE could aid in tracking disease progression and evaluating the effectiveness of therapeutic interventions. This observation is clinically relevant, as it highlights the sensitivity of C-SWE in detecting subtle changes in muscle elasticity that may vary based on baseline muscle tones.

The ability of C-SWE to visualize and quantify changes in muscle elasticity in real time without relying on an acoustic radiation force offers significant advantages over conventional techniques. Its safety profile and cost-effectiveness make it particularly suitable for bedside applications and intraoperative monitoring. Moreover, unlike other imaging modalities, C-SWE allows for the continuous assessment of shear wave propagation, enabling reliable measurements under dynamic conditions.

### 4.3. Differences in the Production of Shear Waves Between High-Intensity Acoustic Pulses and Mechanical Excitation

In the method utilized by Saito et al. [27], acoustic radiation force impulse (ARFI) technology produces shear waves using high-intensity acoustic pulses delivered into the tissue. However, this approach has certain limitations. The use of intense acoustic energy carries the risk of localized tissue heating, particularly near structures such as bones. Furthermore, ARFI-based methods require specialized high-frame-rate ultrasound systems to detect and measure shear wave propagation, which can increase system complexity and cost. These factors may limit the applicability of ARFI methods in certain clinical settings, especially in bedside or intraoperative environments.

In contrast, our C-SWE method employs mechanical excitation to continuously generate shear waves. This approach avoids the use of high-intensity acoustic pulses, thereby ensuring a safer and more tissue-friendly alternative, particularly for living tissues subjected to prolonged monitoring. In addition, C-SWE leverages standard ultrasound systems equipped with a color Doppler functionality, allowing for real-time visualization and quantitative analysis of shear wave propagation. The simplicity and cost-effectiveness of C-SWE make it versatile, enabling its use not only in medical rooms but also in settings such as physical therapy, sports medicine, and intraoperative monitoring.

Moreover, the continuous mechanical excitation employed in C-SWE allows for the direct observation of shear wave propagation, facilitating reliable measurements over time. This feature is particularly advantageous under dynamic conditions such as rocuronium-induced neuromuscular relaxation, as demonstrated in our study. In comparison, ARFI-based methods produce discrete transient shear waves, which may limit their ability to continuously assess muscle elasticity.

The results of our study further underscore the utility of C-SWE in detecting subtle changes in muscle elasticity under controlled conditions, such as general anesthesia. By simultaneously combining SWV measurements and TOF monitoring, we objectively evaluated the relationship between muscle tone and elasticity in a safe and reproducible manner.

### 4.4. Limitations

Nevertheless, this study had some limitations. First, the small sample size (*n* = 11) is a limitation of this study and may restrict the generalizability of our findings. Although 15 patients met the inclusion criteria and were initially enrolled, four were excluded from the analysis due to initial setup errors during the measurement. These errors included issues such as the predefined ROI for Doppler images not matching the depth of the target muscle. While adjustments to the ROI were attempted during measurement, these deviations from the software’s constraints rendered the analysis unusable. Despite this limitation, the exploratory nature of this study and the novel use of simultaneous assessments with C-SWE and TOF monitoring provide valuable preliminary insights into the relationship between muscle tone and elasticity. Larger-scale studies with diverse populations are needed to validate these findings and further explore their clinical implications. Additionally, another limitation related to the analysis software itself was the inability to adjust the predefined ROI. Importantly, newer versions of the software now allow for adjustable ROIs, which could address these challenges in future studies, enhancing both flexibility and measurement accuracy. Second, individual differences in muscle architecture, such as fat infiltration or fiber composition, were not assessed in this study. While BMI was measured as a general indicator of body composition, it does not account for specific muscle and fat characteristics that may influence SWV values. As highlighted by Saito et al. [27], subcutaneous fat thickness can influence SWV values. Common methods for assessing fat thickness include ultrasound imaging, MRI or CT scans, and skinfold calipers. Future studies incorporating these measurements, particularly using ultrasound because of its practicality, could improve the accuracy and clinical relevance of SWV evaluations. Third, a relatively low dose of rocuronium was administered in two increments. Although this approach allowed us to monitor gradual changes in muscle elasticity, it may not reflect the effects of deeper neuromuscular blockades achieved with higher doses. Future studies should consider dose–response relationships to further elucidate the relationship between neuromuscular relaxation and the SWV. Finally, SWV measurements were taken every 5.54 s due to the recording of color Doppler images for several seconds and the video processing of the recorded images [15], and TOF measurements were taken every 15 s, which did not allow for perfect temporal synchronization. Although this did not significantly affect the results because the data were compared before and after rocuronium administration, this would be a problem when analyzing correlations in real time. Further improvements are needed.

## 5. Conclusions

In conclusion, our study demonstrated that C-SWE is a reliable and sensitive method for evaluating muscle elasticity during general anesthesia. By combining SWV measurements, which reflect muscle elasticity, with TOF monitoring, which assesses muscle tone, we comprehensively evaluated muscle relaxation and mechanical properties. These findings have significant implications for the intraoperative evaluation of muscle function and may pave the way for broader applications of C-SWE in musculoskeletal medicine.

## Figures and Tables

**Table 1:** Baseline characteristics of the patients.

	Mean	SEM
Age (y)	59.4	4.6
Sex	Male: 7, Female: 4	
Height (cm)	163.4	2
Weight (kg)	70.1	3.7
BMI (kg/m^2^)	26.3	1.3

**Table 2:** Correlation between demographic data and shear wave velocity for each timing and change.

	Shear Wave Velocity (m/s)		
	Before Rocuronium Administration	Complete Muscle Relaxation	Change
Age (y)	r = −0.101, *p* = 0.767	r = 0.285, *p* = 0.396	r = 0.422, *p* = 0.197
Sex	r = 0.282, *p* = 0.401	r = 0.102, *p* = 0.766	r = −0.301, *p* = 0.368
Height (cm)	r = −0.347, *p* = 0.296	r = −0.303, *p* = 0.365	r = 0.197, *p* = 0.561
Weight (kg)	r = 0.007, *p* = 0.984	r = −0.404, *p* = 0.218	r = −0.404, *p* = 0.218
BMI (kg/m^2^)	r = 0.174, *p* = 0.609	r = −0.280, *p* = 0.405	r = −0.521, *p* = 0.101

r: correlation coefficient.

## Data Availability

The data supporting the findings of this study are available from the corresponding author (H.S.) upon request. The data were not publicly available because they contained information that could compromise the privacy of the participants.
